# Methodological challenges in surgical randomized trials: systematic review of rectal cancer studies

**DOI:** 10.1093/bjsopen/zrag102

**Published:** 2026-09-21

**Authors:** Daichi Kitaguchi, Mariano Giménez, Antonello Forgione, Tatsuya Oda, Jacques Marescaux

**Affiliations:** Research Institute Against Digestive Cancer (IRCAD), Strasbourg, France; Department of Gastrointestinal and Hepatobiliary and Pancreatic Surgery, Institute of Medicine, University of Tsukuba, Tsukuba, Japan; Research Institute Against Digestive Cancer (IRCAD), Strasbourg, France; DAICIM Foundation, Buenos Aires, Argentina; Research Institute Against Digestive Cancer (IRCAD), Strasbourg, France; Department of Gastrointestinal and Hepatobiliary and Pancreatic Surgery, Institute of Medicine, University of Tsukuba, Tsukuba, Japan; Research Institute Against Digestive Cancer (IRCAD), Strasbourg, France

**Keywords:** surgical procedures, operative, randomized clinical trial, bias, GRADE approach, rectal neoplasms

## Abstract

**Background:**

Surgical randomized clinical trials (RCTs) have structural features that differ fundamentally from those of non-surgical trials, which may complicate appraisal using standard assessment frameworks. This methodological systematic review used rectal cancer surgery RCTs as a high-complexity exemplar to identify surgically specific methodological challenges and to examine their interaction with established tools for assessing risk of bias and certainty of evidence.

**Methods:**

A systematic literature search was conducted in PubMed, Embase, and the Cochrane Library on 1 November 2025. Eligible studies were RCTs evaluating surgery-related interventions for rectal cancer in adult patients, published in English from 2020 onward, with sufficient methodological detail. Included trials were categorized into predefined, mutually exclusive surgical RCT types. Methodological features were descriptively summarized and conceptually mapped to the domains of the revised Cochrane Risk of Bias (RoB 2) tool and the GRADE framework using both quantitative tabulation and narrative synthesis.

**Results:**

Of 1529 records identified, 45 publications reporting on 35 RCTs were included. Trials were classified into five surgical RCT categories. Surgically specific methodological challenges were most frequently observed in RoB 2 domains D2 (bias due to deviations from intended interventions) and D4 (bias in measurement of the outcome), as well as in the GRADE domain of indirectness.

**Conclusion:**

This review suggests that several challenges encountered when applying RoB 2 and GRADE to surgical RCTs arise from structural characteristics of surgical trial design rather than obvious methodological shortcomings. Contextualizing standard appraisal frameworks within the realities of surgical research may allow for a more nuanced interpretation of existing evidence, inform future trial design, and support more appropriate evidence synthesis.

## Teaser

Surgical randomized clinical trials (RCTs) present unique methodological challenges that are not fully captured by standard appraisal tools. In a systematic review of 35 rectal cancer surgery RCTs, key issues were most prominent in biases related to deviations from intended interventions, outcome measurement, and indirectness. These findings suggest that many apparent limitations reflect inherent features of surgical research, highlighting the need for more context-sensitive interpretation of evidence quality.

## Introduction

Randomized clinical trials (RCTs) are central to evidence-based medicine, providing the most reliable estimates of treatment effects when rigorously designed and conducted^1,2^, and meta-analyses synthesizing such trials are regarded as the highest level of clinical evidence. However, despite advances in reporting and transparency reflected in the CONSORT statement^3^, methodological limitations may result in a high risk of bias in individual trials and reduced certainty of the synthesized evidence. Subsequently, rigorous assessment of these aspects remains essential to preserve the credibility of evidence-based practice.

Despite the availability of robust appraisal frameworks such as the revised Cochrane Risk of Bias (RoB 2) tool and GRADE^4^, surgical RCTs differ fundamentally from trials of medical, pharmaceutical, and other non-procedural interventions, making appropriate appraisal a challenging task. Procedural complexity, operator-dependent performance, and context-specific factors make surgical interventions difficult to fully standardize or control. Although these features do not necessarily represent methodological deficiencies, they nonetheless complicate interpretation for researchers, reviewers, and guideline developers.

A clearer understanding of the structural features inherent to surgical RCTs and of their interactions with existing appraisal frameworks is essential for fair evaluation of surgical evidence and for informing the design of future studies. In this methodological systematic review, rectal cancer surgery RCTs were used as a high-complexity exemplar in which methodological challenges of surgical trials are particularly apparent.

## Methods

### Study design

This systematic review was conducted in accordance with the PRISMA 2020 guidelines^5^. A prospective protocol was registered in PROSPERO (registration no. CRD420251269754) before data extraction was initiated. Rectal cancer was used as a technically complex surgical model for examining methodological characteristics of surgical RCTs.

### Search strategy

A comprehensive literature search was conducted in PubMed, Embase, and the Cochrane Library. A core search strategy was developed using a combination of free-text terms and controlled vocabulary related to rectal cancer, surgical intervention types, and randomized trial design. The base free-text search string was as follows: (rectal cancer OR rectal carcinoma) AND (laparoscop* OR robot* OR proctectomy OR ‘total mesorectal excision’ OR TME OR TaTME OR anastomo*) AND (‘randomized controlled trial’ OR randomized OR randomized). This core strategy was subsequently adapted for each database by incorporating the appropriate controlled vocabulary (for example, MeSH terms in PubMed and Emtree terms in Embase) and applying database-specific syntax. Complete search strategies for all databases are provided in *Table S1*. The search was restricted to human studies published in English, and the final search update was performed in November 2025.

### Eligibility criteria

Studies were eligible if they met all the following criteria: RCTs evaluating surgery-related interventions for rectal cancer in adult patients; sufficient methodological detail to allow for the assessment of surgically specific design features; and full-text peer-reviewed articles published in English from 2020 onward. Studies were excluded if they were review articles, editorials, letters, conference abstracts, or other publications not reporting original research, non-randomized designs, or lacked extractable methodological information. Studies primarily evaluating neoadjuvant or adjuvant therapies rather than surgical techniques were also excluded.

### Study selection

All records retrieved from the search were screened by one author (D.K.) based on titles and abstracts using predefined eligibility criteria, and all screening decisions were independently verified by a second author (M.G.). Studies deemed potentially eligible proceeded to full-text assessment. Disagreements at any stage were resolved through discussion and consensus. When multiple publications reported data from the same trial population, the report containing the most complete methodological information was selected to avoid duplication of extracted data. Reasons for exclusion at the full-text stage were documented.

### Data extraction

Primary data extraction was performed by one author (D.K.) using a structured extraction form, and all extracted data were independently verified by a second author (M.G.). Extracted variables included general trial characteristics (for example, author, year, country, study setting, comparison, sample size, study period, and population), as well as key methodological features relevant to surgical RCTs (for example, surgeon or centre requirements, primary endpoint, crossover and conversion to alternative surgical approaches, and compliance with patient-reported outcomes (PROs)). Disagreements were resolved through discussion and consensus. Missing information was recorded as reported in the original publications, and no data were imputed.

### Data synthesis

Methodological features were summarized descriptively using frequencies and proportions. Extracted surgical RCTs were first defined and categorized to ensure mutually exclusive and conceptually consistent classifications. Studies were grouped into predefined categories reflecting different surgical trial contexts. Methodological items were coded using simple and inclusive definitions; a feature was considered present when the publication provided any explicit description relevant to that construct. In addition to quantitative tabulation, a narrative synthesis was performed in which each methodological feature was conceptually mapped to relevant domains of the RoB 2 tool and to GRADE dimensions related to the certainty of evidence.

## Results

### Study selection

**Figure 1:**
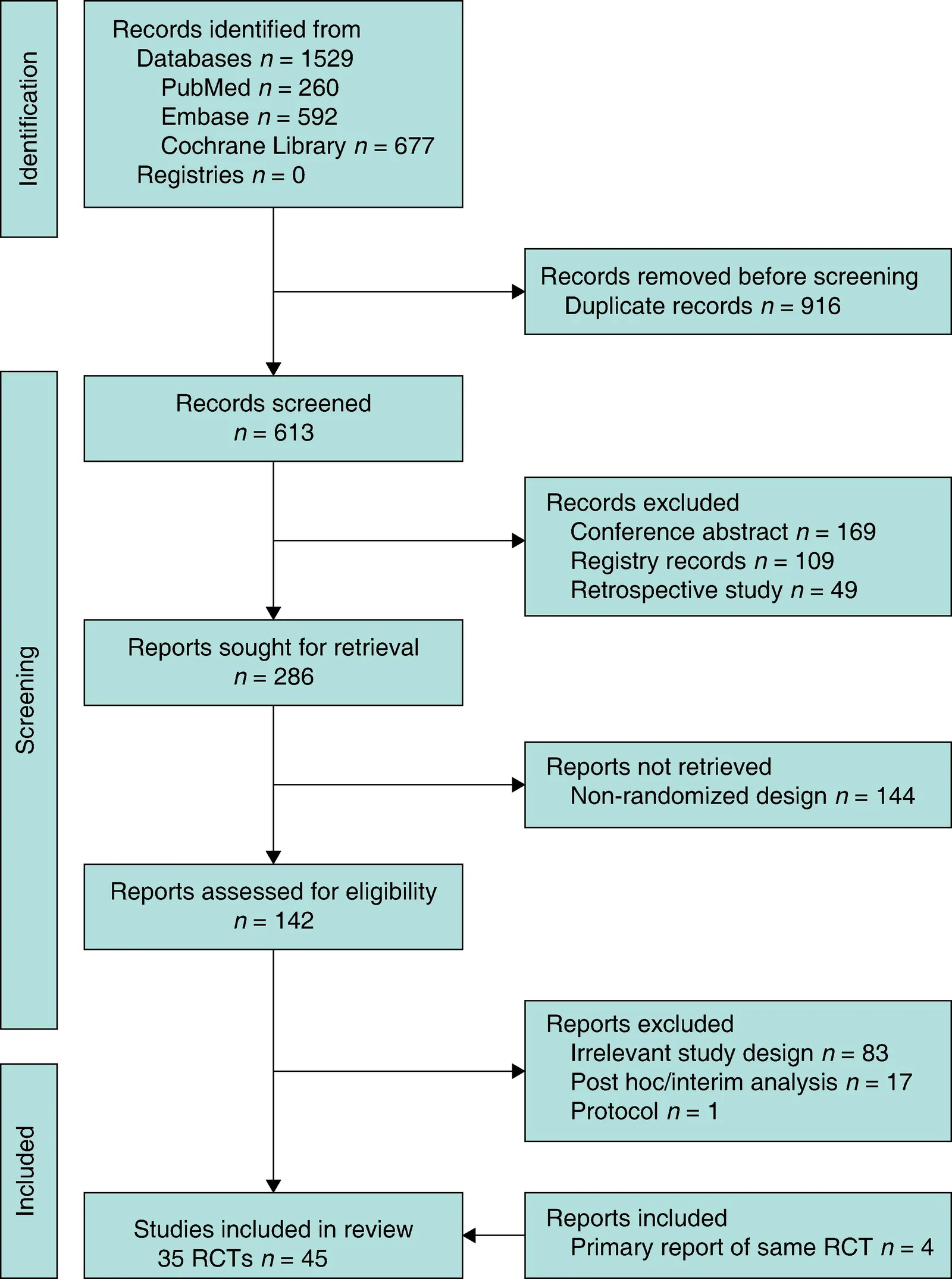
PRISMA flow diagram for the selection of RCTs for inclusion in this study RCT, randomized clinical trial.

*Figure 1* shows the PRISMA flow diagram for study selection. In all, 1529 records were identified through database searches: 260 from PubMed, 592 from Embase, and 677 from the Cochrane Library. After the removal of 916 duplicate records, 613 unique records remained. Of these, 169 conference abstracts, 109 registry records, and 49 retrospective studies were excluded, leaving 286 records for title and abstract screening. During this screening phase, 144 articles were excluded because they were not RCTs. Consequently, 142 full-text articles were assessed for eligibility. Of these, 83 articles were excluded due to an inappropriate study design, 17 were excluded because they reported post hoc or interim analyses, and one was excluded because it was a protocol paper. Of the remaining 41 publications, several reported only long-term follow-up outcomes. As a result, four additional primary publications reporting the corresponding short-term findings from the same trial populations were included. Ultimately, 45 publications^6–50^ corresponding to 35 RCTs were included in this systematic review.

### Study classification

*Table 1* summarizes the classification framework used for surgical RCTs. Each category was defined by a fixed element, whereas the compared element varied within that constraint. By defining one key component of the surgical intervention, comparisons were structured so that each RCT category was mutually exclusive, with no overlap across categories. All included surgical RCTs were classified into five categories: surgical approach RCTs, comparing different surgical approaches for the same operation; surgical strategy RCTs, comparing different operative strategies within the same operation; surgical procedure RCTs, comparing different procedures for the same disease setting; anastomosis/extraction technique RCTs, comparing different reconstruction or specimen extraction methods following the same resection; and surgical technology RCTs, comparing the same operation performed with and without specific surgical technologies.

**Table 1:** Classification framework for included surgical RCTs

RCT category	Description	Fixed element	Compared element	Temporal trends in rectal cancer surgery	No. of included RCTs
Surgical approach RCT	RCTs comparing different surgical approaches for the same operation	Operation	Access route or operative platform	Shift from laparoscopic to robotic surgery TaTME exploration in selected centres	11
Surgical strategy RCT	RCTs comparing different surgical strategies within the same operation	Operation	Operative plan or principle	MIS enabling refinement of dissection strategies Increased focus on preservation of urogenital function	7
Surgical procedure RCT	RCTs comparing different procedures for the same disease setting	Disease setting	Type of operation	Transition from CRT to TNT Growing interest in organ preservation	4
Anastomosis/extraction technique RCT	RCTs comparing different reconstruction or extraction methods after the same resection	Resection	Reconstruction or specimen extraction method	Renewed interest in TCA and emergence of TTSS NOSE as an available specimen extraction option	5
Surgical technology RCT	RCTs comparing the same operation with and without specific surgical technologies	Operation	Device use	Rapid adoption of new surgical technologies, including ICG fluorescence imaging	8

RCT, randomized clinical trial; TaTME, transanal total mesorectal excision; MIS, minimally invasive surgery; CRT, chemoradiotherapy; TNT, total neoadjuvant therapy; TCA, Turnbull–Cutait anastomosis; TTSS, transanal transection and single-stapled anastomosis; NOSE, natural orifice specimen extraction; ICG, indocyanine green.

### Characteristics of the included surgical RCTs

*Table S2* presents the characteristics of the included surgical RCTs in rectal cancer. In all, 35 surgical RCTs in rectal cancer were included and classified into five categories: surgical approach RCTs (11), surgical strategy RCTs (7), surgical procedure RCTs (4), anastomosis/extraction technique RCTs (5), and surgical technology RCTs (8). Overall, among the 35 included surgical RCTs, 14 were single-centre studies, 18 were multicentre studies conducted within a single country, and 3 involved international multicentre collaboration. Trial sample sizes ranged from 40 to 1171 participants, with a median of 163 (interquartile range 93.5–343.5) participants.

### Methodological challenges across RoB 2 domains

*Table 2* presents a conceptual mapping of RoB 2 domains to methodological challenges commonly encountered in surgical RCTs, with illustrative examples from the included studies. The following sections outline key methodological challenges across individual RoB 2 domains.

**Table 2:** Mapping RoB 2 domains to surgical RCT-specific challenges and examples

RoB 2 domain	Surgical challenge	Bias mechanism	Surgical RCT design	Key example
D1: Bias arising from the randomization process	No major surgery-specific challenges identified			
D2: Bias due to deviations from intended interventions	Inability to blind surgical interventions	Patient expectation → non-adherence	Surgical approach RCTs/surgical procedure RCTs	Crossover rates: MIS *versus* open 4.8% (75 of 1550); LE *versus* TME 16.4% (61 of 373)
	Inability to blind surgical interventions	Clinician-dependent intervention delivery	All surgical RCT designs	
	Non-randomization of surgeons	Operator-dependent performance	Surgical approach RCTs/surgical strategy RCTs	Surgeon experience criteria reported in 13 of 35 trials (37%)
	Conversion during surgery	Post-randomization crossover	Surgical approach RCTs	Conversion to open surgery contributes to crossover
	Procedural constraints	Asymmetric crossover → directional bias	Specific surgical RCT designs with asymmetric conversion	Asymmetric crossover (for example, MIS → open unavoidable; reverse not feasible)
D3: Bias due to missing outcome data	Incomplete PRO compliance	Missing data/selective reporting	Surgical RCTs including PROs	Mean PRO compliance 73.8%, declining over follow-up
D4: Bias in measurement of the outcome	Inability to blind surgical interventions	Differential outcome assessment	Surgical RCTs with perioperative outcome measures	Clinical endpoints used as primary outcomes in 12 of 35 trials (34%)
	Inability to blind patients	Subjective outcome assessment	Surgical RCTs including PROs	PROs reported in 16 of 35 trials (46%), including 8 (23%) as primary endpoints
D5: Bias in selection of the reported results	No major surgery-specific challenges identified			

RoB 2, revised Cochrane risk-of-bias tool for randomized trials; RCT, randomized clinical trial; MIS, minimally invasive surgery; LE, local excision; TME, total mesorectal excision; PRO, patient-reported outcome.

#### D1: bias arising from the randomization process

No major methodological challenges unique to surgical RCTs were identified for bias arising from the randomization process, because standard randomization methods, including sequence generation and allocation concealment, are generally applicable across clinical trial designs.

#### D2: bias due to deviations from intended interventions

In surgical RCTs, deviations from intended interventions represent a major source of potential bias, largely driven by the inherent inability to blind surgical procedures, which prevents concealment of treatment allocation at both patient and provider levels and increases the risk of post-randomization deviations. The lack of blinding may influence patient expectations and treatment preferences, contributing to systematic non-adherence to allocated interventions and compromising treatment fidelity. Observed crossover patterns further demonstrate that randomization alone does not ensure adherence to assigned interventions in surgical settings.

In addition to patient-related factors, clinician behaviour is also affected by the inability to blind surgical interventions. Although randomization is applied to patients, surgeons cannot be randomized, and surgical performance remains inherently operator dependent. This results in systematic variability in intervention delivery, contributing to performance bias. Given the recognized influence of surgeon expertise on perioperative and oncological outcomes, this finding highlights substantial variation in the reporting of measures intended to address provider-level heterogeneity.

Conversion during surgery represents a structural source of deviation unique to surgical trials and may directly undermine adherence to allocated interventions. Importantly, such deviations are often asymmetric, reflecting procedural constraints rather than random variation, and may subsequently introduce directional bias in estimated treatment effects. This asymmetry further complicates the interpretation of intention-to-treat analyses.

Crossover or conversion events were reported in 33 of 35 included RCTs, although the frequency and clinical significance of these events varied substantially between studies. These findings indicate that deviations from intended interventions are common and may reflect structural challenges in surgical RCTs, with potential implications for internal validity and the interpretation of treatment effects.

#### D3: bias due to missing outcome data

Postoperative functional outcomes and quality of life (QoL) are clinically essential endpoints in rectal cancer surgery. These outcomes are commonly captured as PROs. However, incomplete data collection and suboptimal compliance are frequent challenges in surgical RCTs. Lower PRO compliance may result in missing outcome data, potentially leading to underestimation of functional impairment.

**Figure 2:**
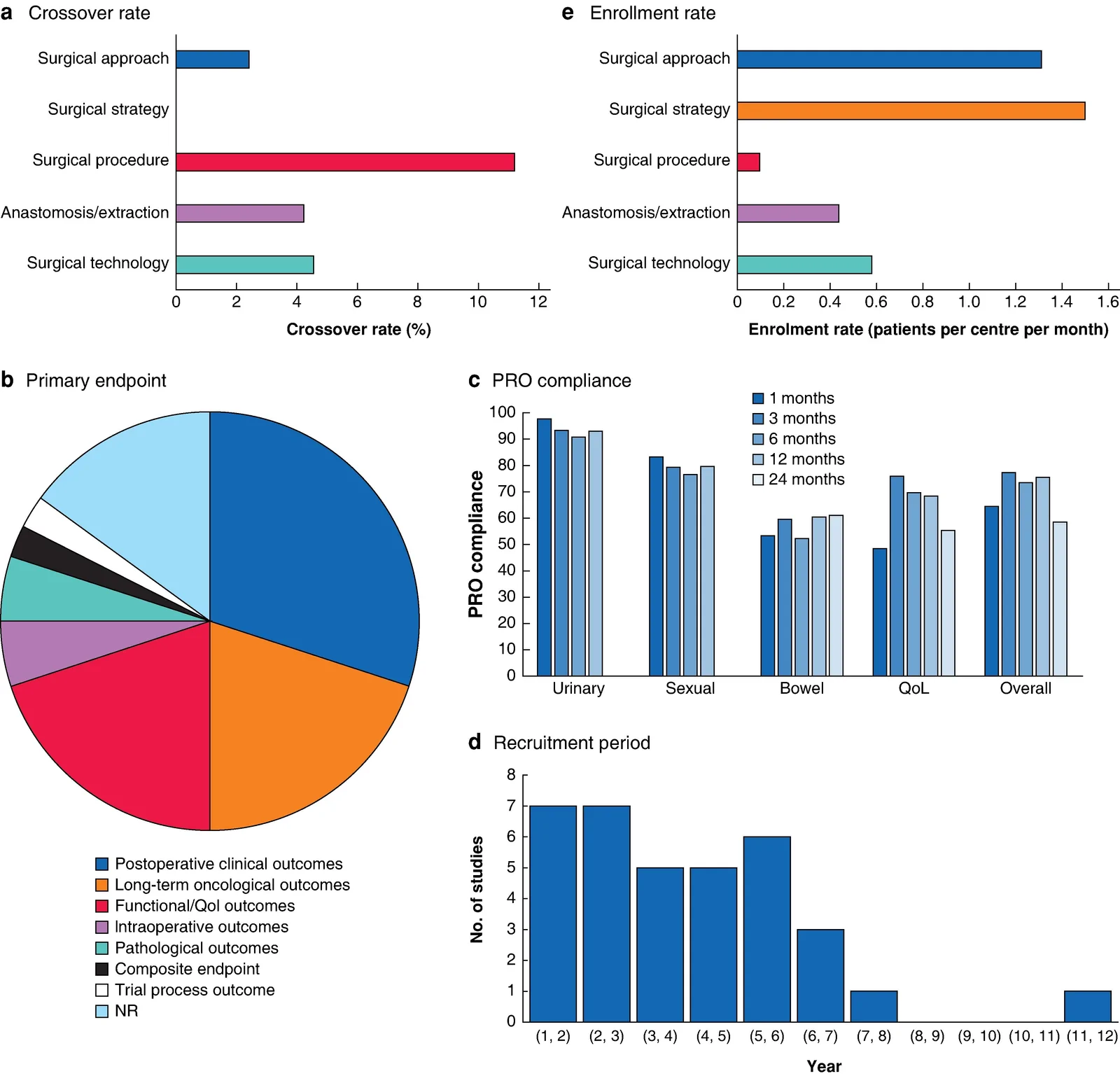
Methodological characteristics across included surgical RCTs **a** Crossover rates per trial category. **b** Primary endpoint distribution. **c** Temporal and domain-specific PRO compliance. **d** Recruitment duration. **e** Enrolment rates. RCTs, randomized clinical trials; QoL, quality of life; NR, not reported; PRO, patient-reported outcome.

*Figure 2b* illustrates the primary endpoints of the 35 included RCTs. Sixteen trials reported PROs, and functional or QoL outcomes were designated as the primary endpoint in eight RCTs. Among the 16 trials reporting PROs, overall PRO compliance was 73.8%. As shown in *Fig. 2c*, PRO compliance varied over time, peaking at 77.3% at 3 months after surgery and declining to 58.5% at 24 months. Compliance also differed across PRO domains. Urinary function was the most frequently reported PRO and demonstrated the highest compliance rate (92.7%). In contrast, more sensitive PRO domains, such as sexual function, bowel function, and overall QoL, showed a substantially lower compliance, with a mean compliance rate of 66.7%. The progressive decline in response rates over time highlights the practical challenges of long-term collection of PROs in surgical trials and may contribute to uncertainty in estimates of functional outcomes.

#### D4: bias in measurement of the outcome

In surgical RCTs, outcome measurement is closely influenced by the inability to blind surgical interventions. Lack of blinding was associated with differential outcome assessments, particularly in trials adopting perioperative or short-term clinical outcomes as primary endpoints, compared with trials focusing on long-term oncological outcomes, which are generally more objective. The inability to blind patients was also associated with a greater subjectivity in outcome measurement, especially in RCTs using PROs. When patients were aware of treatment allocation, symptom reporting and functional assessments showed a greater variability, indicating an increased susceptibility of PRO-based endpoints to measurement-related bias.

As shown in *Fig. 2b*, only 8 of the 35 included RCTs designated long-term oncological outcomes as their primary endpoint, whereas 12 RCTs adopted postoperative clinical outcomes, such as postoperative complications, as primary endpoints. Although postoperative complications were commonly graded using the Clavien–Dindo classification, this therapy-oriented classification may be less robust in unblinded settings, where clinical decision-making may influence both detection and grading. Other reported endpoints included intraoperative outcomes such as conversion to open surgery and anal preservation, which served as the primary endpoint in 2 of 35 trials, and pathological outcomes such as the number of harvested lymph nodes and quality of total mesorectal excision, which were primary endpoints in 2 of 35 trials. These outcomes were influenced by intraoperative judgment and postoperative assessment and subsequently subject to variability in unblinded surgical RCTs.

#### D5: bias in selection of the reported results

No methodological challenges specific to surgical RCTs were identified for bias related to selection of the reported results. Issues such as selective outcome reporting and outcome switching are not unique to surgical trials and are similarly encountered across clinical trial designs.

### Methodological challenges across GRADE domains

*Table 3* presents a conceptual mapping of GRADE domains to methodological challenges commonly encountered in surgical RCTs, together with illustrative examples. The following sections describe surgical RCT-specific challenges relevant to key GRADE domains, including inconsistency, indirectness, and imprecision.

**Table 3:** Mapping GRADE domains to surgical RCT-specific challenges and examples

GRADE domain	Surgical RCT-specific challenges	Factors affecting certainty	Representative designs	Examples based on included RCTs
Risk of bias	See Table 2			
Inconsistency	Limited standardization across surgeons or centres	Surgeon of centre variability	Single-centre RCTs	Single-centre RCTs: 14 of 35 (40%)Studies with clearly defined surgeon experience requirements: 13 of 35 (37%)
Indirectness	Extended recruitment	Learning curveTechnical/technological evolution	All	Overall weighted mean recruitment period: 51.2 monthsRecruitment period ≥ 5 years: 11 of 35 trials (31%)Time-related heterogeneity beyond the intended comparison
	Difficulty conducting international trials	Geographical concentration	Single-country RCTsSurgical approach RCTs	Single-country RCTs: 32 of 35 (91%)China: 16 of 35 trials (46%), 5496 of 10 101 participants (54%)No neoadjuvant CRT: 1 of 35 (3%) Mean patient BMI: East Asia 23.3 kg/m^2^, Europe 26.4 kg/m^2^, and North America 28.0 kg/m^2^
	Strict surgeon eligibility	Limited generalizability	Robotic *versus* laparoscopic TME RCTs	REAL trial: ≥ 100 laparoscopic robotic colorectal surgeries per year and ≥ 50 robotic plus ≥ 50 laparoscopic rectal surgeriesCOLRAR trial: > 100 rectal cancer surgeries per year and ≥ 100 robotic or laparoscopic rectal surgeries before the trial.
Imprecision	Low enrolment rate PRO compliance	Reduced effective sample size	Surgical strategy RCTs RCTs with PROs	Overall weighted mean enrolment rate: 0.63 patients per centre per monthSurgical procedure RCTs: 0.11 patients per centre per month Overall PRO compliance: 73.8%

RCT, randomized clinical trial; CRT, chemoradiotherapy; BMI, body mass index; TME, total mesorectal excision; PRO, patient-reported outcome.

#### Inconsistency

In surgical RCTs, inconsistency across studies may arise from limited standardization at the surgeon and centre levels. Variability in surgical expertise, technique, and institutional practice is unavoidable and may be associated with heterogeneity in treatment effects across trials. This variability is more apparent when evidence synthesis includes a high proportion of single-centre RCTs, where interventions are implemented within distinct institutional settings. In contrast, multicentre RCTs with predefined surgeon credentialing or experience requirements reflect a greater procedural standardization. Among the 35 included RCTs, 14 were single-centre studies, and 13 trials reported predefined surgeon experience requirements.

#### Indirectness

In surgical RCTs, patient enrolment challenges, including limited eligibility due to technical difficulty and reluctance related to treatment preferences, often result in prolonged recruitment periods. Extended recruitment increases the likelihood that learning-curve effects, perioperative management, and surgical technology evolve during the study period, leading to chronological heterogeneity beyond the intended intervention comparison. Such temporal heterogeneity introduces indirectness by reducing the comparability of participants and interventions over time and by limiting the applicability of findings to current clinical practice.

Geographical concentration represents an additional source of indirectness in surgical RCTs. Conducting international trials is often challenging due to variations in surgical practice and healthcare systems. Consequently, most trials are conducted within a single country. This concentration may introduce systematic differences in patient populations and healthcare contexts, restricting the generalizability of findings. In rectal cancer surgery, neoadjuvant treatment strategies and patient body mass index are key determinants of operative difficulty and outcomes. Regional variation in these characteristics further contributes to indirectness by limiting the transferability of results across populations.

Indirectness also arises at the provider level. Variability in surgeon experience and technical proficiency may contribute to within-study bias and between-study inconsistency, whereas strict surgeon eligibility criteria may reduce generalizability. Highly selective eligibility criteria, although reducing variability, may restrict applicability to high-volume centres and expert surgeons, thereby limiting the external validity of trial findings.

#### Imprecision

**Figure 3:**
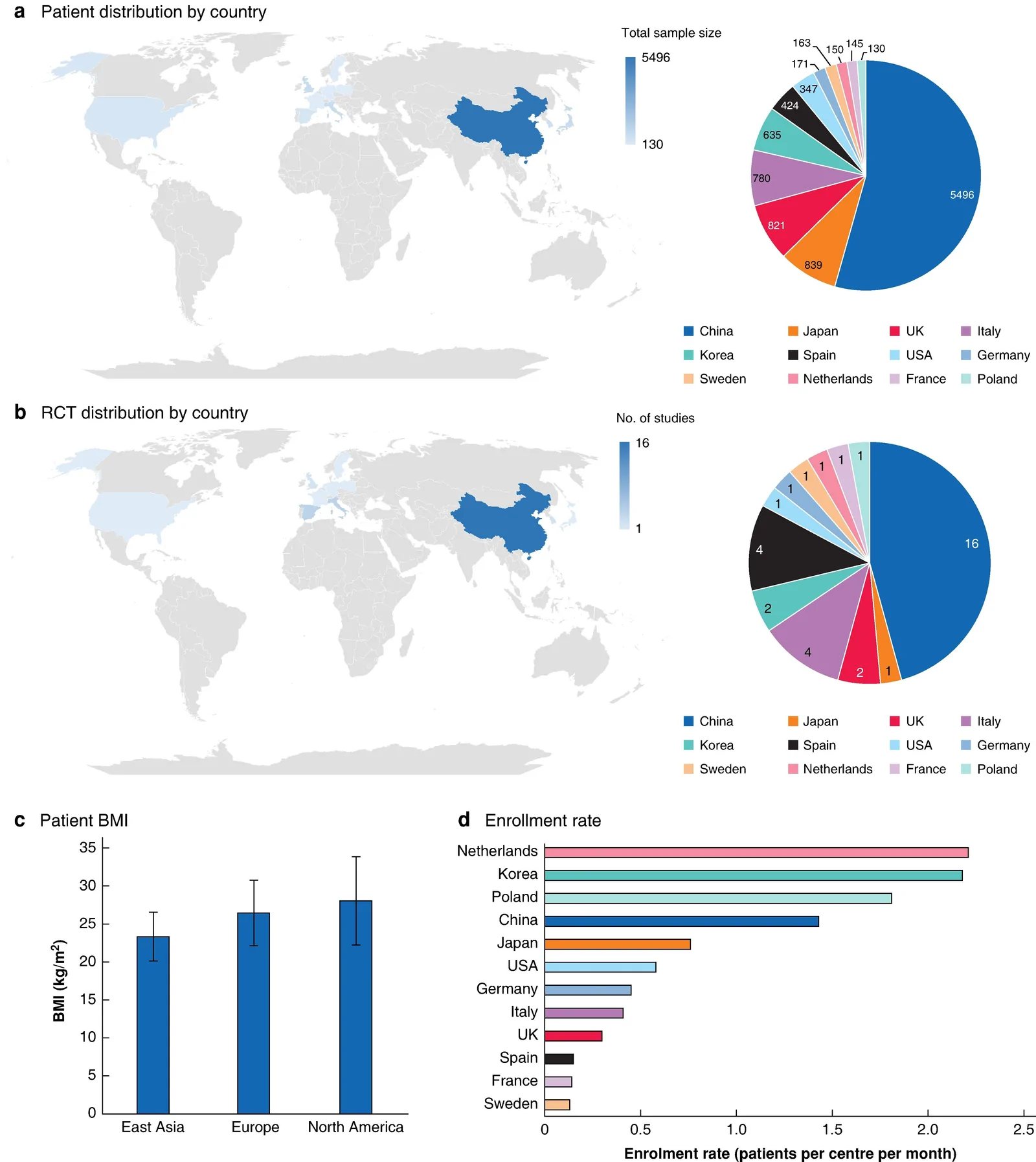
Geographical characteristics across included surgical RCTs **a** Distribution of enrolled patients by country. **b** Distribution of included RCTs by country. **c** Regional differences in the mean BMI of enrolled patients. Error bars indicate the standard deviation. **d** Enrolment rates by country. RCTs, randomized clinical trials; BMI, body mass index.

In surgical RCTs, low enrolment rates represent a major challenge that can lead to imprecision by limiting the effective sample size. As shown in *Fig. 2e*, the overall weighted mean enrolment rate across the 35 included RCTs was 0.63 patients per centre per month. Enrolment was particularly slow in surgical procedure RCTs, where large differences between treatment arms may hinder patient willingness to participate; in this category, the weighted mean enrolment rate was 0.11 patients per centre per month. Substantial variation in enrolment efficiency was also observed across countries (*Fig. 3d*). Mean enrolment rates exceeded 2.1 patients per centre per month in the Netherlands and Korea, whereas rates below 0.2 patients per centre per month were observed in Sweden, France, and Spain. In addition to slow recruitment, incomplete outcome ascertainment further contributes to imprecision. Suboptimal compliance with PROs reduces the effective sample size available for analysis, thereby increasing uncertainty around estimated treatment effects.

## Discussion

Surgical RCTs face methodological challenges that differ fundamentally from those in other medical fields. In this methodological systematic review of 45 articles reporting on 35 RCTs in rectal cancer surgery, trials were classified into five non-overlapping categories and trial-level data were extracted to identify challenges specific to surgical RCTs. Using this framework, RoB 2 and GRADE domains were mapped to surgically specific methodological issues, with illustrative examples from included studies. Structural methodological challenges were most frequently observed in RoB 2 domains D2 and D4, as well as in the GRADE domain of indirectness. Importantly, these challenges do not indicate poor trial quality, but rather reflect inherent features of surgical trial design and conduct. Recognizing such realities is critical for appropriate interpretation, evidence synthesis, and future trial design.

The inability to blind surgical interventions and the impracticality of sham or placebo surgery constitute a fundamental challenge unique to surgical RCTs. Consequently, clinician and patient expectations or preferences may contribute to deviations from intended interventions and complicate patient enrolment. Conversion surgery, a surgery-specific phenomenon often associated with crossover, poses additional challenges for trial design and interpretation. In addition, surgical RCTs frequently assess postoperative outcomes such as complications, length of hospital stay, and functional or QoL measures in addition to long-term oncological endpoints. Although clinically meaningful, these outcomes are often less objective than survival outcomes and may be susceptible to bias due to therapy-oriented definitions or reliance on PROs.

Surgical fields are particularly susceptible to rapid temporal shifts. Rectal cancer surgery provides a useful high-complexity example of this phenomenon, because multiple practice-changing developments may occur over relatively short time frames, including transitions from laparoscopic to robotic approaches^51^, shifts in neoadjuvant treatment strategies from chemoradiotherapy to total neoadjuvant therapy^52,53^, increasing interest in organ-preserving strategies^54,55^, adoption of novel anastomotic techniques^56^, and the rapid introduction of new surgical technologies^57^. Such evolving clinical contexts may introduce chronological bias beyond the intended intervention comparison. Consequently, prolonged recruitment in rapidly evolving surgical settings may compromise both internal and external validity by introducing time-dependent heterogeneity, underscoring the importance of efficient and timely patient enrolment in surgical RCTs.

In the present review, trials conducted in the Netherlands demonstrated the highest enrolment efficiency (*Fig. 3d*), suggesting that specific design features may help mitigate time-related methodological challenges. The SPONGE trial^43^ was conducted as a single-centre RCT embedded within a nationwide Dutch colorectal cancer cohort^58^ using a Trials within Cohorts (TwiCs) design^59,60^. By integrating randomization within an established cohort and using staged informed consent, eligible patients could be systematically identified and approached, enabling rapid and efficient enrolment. This approach facilitated timely completion of recruitment, resulting in a high proportion of eligible patients being enrolled and illustrating one pragmatic strategy to address enrolment-related challenges in rapidly evolving surgical RCTs.

Together, the findings of this study highlight areas in which surgery-specific methodological guidance may be beneficial. Such guidance could complement existing frameworks, including RoB 2 and GRADE, by providing additional context for structural features commonly encountered in surgical trials.

Based on the findings, several key directions are proposed to improve the design and evaluation of surgical RCTs. First, future methodological research should evaluate whether explicit distinction between structural constraints inherent to surgical trials and avoidable methodological shortcomings could improve risk-of-bias and certainty-of-evidence assessments. If shown to be beneficial, such distinctions could inform future adaptations of existing appraisal frameworks. A trial-design-specific and stratified adaptation of existing appraisal frameworks could help maintain methodological rigour and ensure transparent evaluation of surgical evidence. Second, future surgical trial design should adopt pragmatic strategies, which directly address recruitment inefficiency and preference-related bias. For example, randomized trials embedded within prospective cohorts may improve feasibility and external validity in rapidly evolving surgical contexts. Third, the development of additional reporting guidance specific to surgical trials may improve transparency and reproducibility. This should include *a priori* definition of surgeon experience criteria, explicit handling of conversion and crossover in the analysis, and clear temporal contextualization of recruitment periods. The adoption of such recommendations, ideally formalized through an international multidisciplinary consensus process, could well provide the foundation for modern surgical research guidelines that more accurately reflect the complexity of surgical interventions while preserving the core principles of evidence-based medicine.

This review has several limitations. Analyses were based on trial-level reporting, and individual patient-level data were not available. Screening and extraction were performed by a primary reviewer with independent verification rather than duplicate independent review. Methodological challenges were identified through descriptive and conceptual synthesis rather than formal grading or quantitative pooling of effect estimates. Finally, this review was restricted to RCTs in rectal cancer surgery, which represents a particularly complex and technically demanding area of surgical practice, and methodological challenges in other surgical specialties were not directly examined. Although several of the identified challenges may reflect structural features of surgical trial design, their generalizability to other surgical specialties should be interpreted with caution. Future studies extending similar analyses to additional surgical specialties may provide a more comprehensive understanding of shared methodological challenges in surgical RCTs.

## Supplementary Material

zrag102_Supplementary_Data

## Data Availability

The data supporting the findings of this study are available from the corresponding author upon reasonable request.
