# Supplementary material for: Methodological challenges in surgical randomized trials: systematic review of rectal cancer studies
Source: BJS Open. 2026 Sep 21;10(5):zrag102. doi: 10.1093/bjsopen/zrag102 (PMC13591274; doi:10.1093/bjsopen/zrag102)
Supplement: Supplementary file 1 [file zrag102_supplementary_data.docx]

**Methodological Challenges in Surgical Randomized Trials: Systematic Review of Rectal Cancer Studies**

Daichi Kitaguchi^1,2^, Mariano Giménez^1,3^, Antonello Forgione^1^, Tatsuya Oda^2^, Jacques Marescaux^1^

^1^	Research Institute against Digestive Cancer (IRCAD), Strasbourg, France

^2^	Department of Gastrointestinal and Hepatobiliary and Pancreatic Surgery, Institute of Medicine, University of Tsukuba, Tsukuba, Ibaraki, Japan

^3^	DAICIM Foundation, Buenos Aires, Argentina

**Corresponding author.**

Mariano Giménez, MD, PhD

IRCAD France, 1 place de l’Hôpital, 67091 Strasbourg Cedex, France

Phone: +33 3 88 11 90 00

Email: mariano.gimenez@ircad.fr

**Supplementary Materials - Index**

| **Supplementary Tables** |  |
| --- | --- |
| Supplementary Table 1 | *Page 2* |
| Supplementary Table 2 | *Page 3-11* |
| **Supplementary Appendixes** |  |
| PRISMA 2020 Checklist | *Page 12-14* |

**Supplementary Tables**

**Supplementary Table 1:** Search strategies for each database

| Database | Date of last search | Search strategy |
| --- | --- | --- |
| PubMed | 2025-11-30 | (“Rectal Neoplasms”[Mesh] OR rectal cancer*[tiab] OR rectal carcinoma*[tiab]) AND (laparoscopy[Mesh] OR “Robotic Surgical Procedures”[Mesh] OR Anastomosis, Surgical[Mesh] OR laparoscop*[tiab] OR robot*[tiab] OR proctectomy[tiab] OR “total mesorectal excision”[tiab] OR TME[tiab] OR TaTME[tiab] OR anastomos*[tiab]) AND ("Randomized Controlled Trial"[pt] OR randomized[tiab] OR randomised[tiab]) NOT (protocol [tiab] OR protocol[pt] OR review[pt] OR "systematic review"[tiab] OR meta-analysis[pt] OR "meta analysis"[tiab] OR comment[pt] OR editorial[pt] OR letter[pt] OR comment[ti] OR editorial[ti]) AND ("2020/01/01"[dp] : "2025/12/31"[dp]) AND English[lang] |
| Embase | 2025-11-30 | (‘rectum neoplasm’/exp OR ‘rectal cancer’:ti,ab OR ‘rectal carcinoma’:ti,ab) AND (‘laparoscopy’/exp OR ‘robot assisted surgery’/exp OR ‘anastomosis’/exp OR laparoscop*:ti,ab OR robot*:ti,ab OR proctectomy:ti,ab OR ‘total mesorectal excision’:ti,ab OR TME:ti,ab OR TaTME:ti,ab OR anastomos*:ti,ab) AND ('randomized controlled trial'/exp OR randomized:ti,ab OR randomised:ti,ab) NOT (protocol:ti,ab OR protocol/it OR review/it OR 'systematic review':ti,ab OR 'meta analysis'/it OR 'meta analysis':ti,ab OR comment/it OR editorial/it OR letter/it OR comment:ti OR editorial:ti) AND [2020-2025]/py AND [english]/lim |
| Cochrane Library | 2025-11-30 | (rectal cancer OR rectal carcinoma) AND (laparoscop* OR robot* OR proctectomy OR "total mesorectal excision" OR TME OR TaTME OR anastomo*) AND (randomized controlled trial OR randomized OR randomised) NOT (protocol OR review OR systematic review OR meta analy* OR meta-analysis OR comment OR editorial OR letter) |

**Supplementary Table 2:** Characteristics of the included surgical RCTs in rectal cancer

| **Author/Year**  **(Country)** | **Setting** | **Comparison** | **Sample Size** | **Study Period** | **Population** | **Surgeon/Center Requirement** | **Primary Endpoint** | **Crossover** |
| --- | --- | --- | --- | --- | --- | --- | --- | --- |
| ***Surgical approach RCTs*** | |  |  |  |  |  |  |  |
| Chang 2023  (China) | Single-center | Robotic vs. Open AR/APR with Hepatectomy | 171 (86/85) | Jan 2016-May 2018 | 18-75 years; AV ≤ 15 cm; cM1 (liver) | > 10 years’ experience in robotic surgery | Complication | 1.8% |
| Feng 2022/2025  (China) | Multicenter (11) | Robotic vs. Lap TME | 1,171 (586/585) | Jul 2016-Dec 2020 | 18-80 years; AV ≤ 10 cm; cT1-3N0-1M0/ycT1-3NXM0 | ≥ 100 MIS cases/year; ≥ 50 robotic + ≥ 50 lap rectal cases | 3-year LR | 1.1% |
| Feng 2022  (China) | Single-center | Robotic vs. Lap APR | 347 (174/173) | Dec 2013-Dec 2016 | 18-75 years; AV ≤ 5 cm; cT1-3N0-1M0/ycT1-3NXM0 | Two experienced surgeons | Complication | 1.2% |
| Jiang 2022/2025  (China) | Multicenter (22) | Lap vs. Open TME | 1,039 (685/354) | Nov 2013-Jun 2018 | 18-75 years; DL ≤ 5 cm; cT1-T4aNXM0; Size ≤ 5 cm | > 30 lap TMEs/year; ≥ 100 lap TMEs | 3-year DFS | 6.7% |
| Kang 2010/  Jeong 2014/  Park 2021  (South Korea) | Multicenter (3) | Lap vs. Open TME | 340 (170/170) | Apr 2006–Aug 2009 | 18-80 years; AV ≤ 9 cm; cT3NXM0 | Seven credentialed surgeons (median 75 prior lap cases) | 3-year DFS | 0.6% |
| Lin 2022  (China) | Single-center | HALS vs. Lap TME | 80 (40/40) | Feb 2011-Jul 2016 | Age NR; AV < 8 cm; cStage I-III | NR | NR | 0% |
| Liu 2023/  Zeng 2025  (China) | Multicenter (16) | TaTME vs. Lap TME | 1089 (544/545) | Apr 2016-Jun 2021 | 18-75 years; below peritoneal reflex; cStage I–III | ≥ 100 lap TME + ≥ 50 TaTME | 3-year DFS + 5-year OS | 0.5% |
| Park 2023  (South Korea) | Multicenter (3) | Robotic vs. Lap TME | 295 (151/144) | Jul 2011-Feb 2016 | ≥ 18 years; AV < 10 cm; cT1-4aNXM0 | > 100 rectal cases/year; ≥ 100 lap/robotic rectal cases | TME quality | 2.8% |
| Serra-Aracil 2023  (Spain) | Multicenter (4) | TaTME vs. Lap TME | 105 (55/50) | Apr 2015-May 2021 | ≥ 18 years; AV ≤ 10 cm; cT1-3N0-1M0 | Public hospitals experienced in rectal cancer care | Conversion | 8.6% |
| Tang 2023  (China) | Single-center | SILS vs. Lap APR | 51 (25/26) | Sep 2020-Sep 2021 | 18-75 years; AV < 3 cm; cT1-4aNXM0; Size < 4 cm | NR | NR | NR |
| Zhang 2023  (China) | Single-center | SILS+1 vs. Lap AR | 198 (99/99) | Apr 2014-Jul 2016 | 18-80 years; Rectum (/Sigmoid); cT1-4aNXM0 | ≥ 100 lap cases; ≥ 10 SILS+1 cases | 3-year DFS | 0% |
| ***Surgical strategy RCTs*** | |  |  |  |  |  |  |  |
| Feng 2021  (China) | Single-center | Lap LAR with high vs. low IMA ligation | 95 (47/48) | Oct 2016-Sep 2018 | 18-75 years; AV 5-15 cm; cStage I-III | 10-year experience | NR | 0% |
| Hong 2021  (China) | Multicenter (3) | Lap TME with CEMP vs. MAP | 506 (256/250) | May 2016-Jul 2020 | > 18 years; AV 4-15 cm; cStage I-III | NR | 3-year OS | 0% |
| Kruszewski 2021  (Poland) | Single-center | Open AR with high vs. low IMA ligation | 130 (65/65) | Apr 2010-Mar 2016 | > 18 years; AV NR; cT1-3NXM0/ycT0-3NXM0 | Seven colorectal-trained oncological surgeons | OS + DFS + CSS | 0% |
| Li 2023  (China) | Single-center | Lap LAR with IMA sheath preserved vs. peeled | 131 (71/70) | Sep 2018-Dec 2020 | ≥ 18 years; AV 5-15 cm; cT1-3N0M0 | One standardized team | NR | 0% |
| Mari 2019/2023  (Italy) | Multicenter (6) | Lap TME with high vs. low IMA ligation | 214 (111/103) | Jun 2014-Dec 2016 | ≥ 18 years; AV ≤ 15 cm; cStage I-III | ≥20 lap cases/year for 5 years | Urogenital function | 0% |
| Wei 2021/  Fang 2023  (China) | Multicenter (11) | Lap TME with DVF preservation vs. resection | 197 (100/97) | Aug 2015-Jul 2019 | 20-71 years (male); AV 6-12 cm; cT1-4 (anterior T1-2)NXM0; no CRT | ≥50 lap TME | Urogenital function | 0% |
| Yang 2025  (China) | Single-center | Lap ISR with vs. without rectal eversion and total extra-abdominal resection | 74 (39/35) | Mar 2024-Mar 2025 | 18-70 years; AV 3–5 cm; cT1-3N1-2M; Size ≤ 5 cm | NR | Anal preservation | 0% |
| ***Surgical procedure RCTs*** | |  |  |  |  |  |  |  |
| Bach 2021  (UK) | Multicenter (21) | SCRT+LE vs. Upfront TME | 55 (27/28) | Feb 2012-Dec 2014 | ≥18 years; AV NR; cT1-2N0M0; Size ≤ 30 mm | NR | Cumulative recruitment of randomization | 20.0% |
| Nikberg 2025  (Sweden) | Binational multicenter (15) | Hartmann vs. intersphincteric APR | 163 (80/83) | Dec 2014-Dec 2021 | ≥18 years; AV 5-15 cm; cStage I-IV | Three surgeons | Complication | 0% |
| Rullier 2017/2020  (France) | Multicenter (15) | CRT+LE vs. CRT+TME | 145 (74/71) | Mar 2007-Sep 2012 | ≥18 years; AV ≤8 cm; cT2–3N0–1; Size ≤ 4 cm; Scar ≤ 2 cm | NR | Composite of death, recurrence, severe morbidity, and side-effects | 24.1% |
| Serra-Aracil 2023/2025  (Spain) | Multicenter (17) | CRT+LE(TEM) vs. TME | 162 (81/81) | Jul 2010-Oct 2021 | ≥ 18 years; AV ≤10 cm; cT2–3abN0M0; Size ≤ 4 cm | Extensive rectal cancer + TES experience | 2-year LR | 8.0% |
| ***Anastomosis/extraction technique RCTs*** | | |  |  |  |  |  |  |
| Altomare 2025  (Italy) | Multicenter (3) | Lap TME with transanal protective ileostomy vs. reinforcement suture | 54 (29/25) | Dec 2015-Sep 2019 | Age NR; AV 3-8 cm; cT2-4aNXMX | Tertiary referral centers | Anastomotic leak | 3.6% |
| Biondo 2020/2024  (Spain) | Binational multicenter (3) | Ultra-LAR with CAA vs. TCA | 92 (46/46) | Jun 2012-Oct 2018 | ≥18 years; AV NR; cStage I-IV | Large experience in rectal cancer + TCA | Complication | 0% |
| Gavaruzzi 2020  (Italy) | Multicenter (19) | LAR with J-pouch vs. Straight CRA | 379 (190/189) | Oct 2009-Feb 2016 | ≥ 18 years; AV ≤ 11 cm; cStage I-III | NR | QoL and bowel function | 3.7% |
| Planellas 2020  (Spain) | Single-center | Lap AR with end-to-end vs. side-to-end anastomosis | 65 (35/30) | Apr 2016-Oct 2017 | ≥ 18 years; Rectum (/Sigmoid); cStage I-IV | Experienced lap colorectal surgeon | Anorectal function | 0% |
| Zhou 2020  (China) | Single-center | Lap AR with vs. without NOSE | 241 (122/119) | Jun 2016-May 2019 | ≥ 18 years; Rectum (/Sigmoid); cStage I-III | NR | NR | 8.0% |
| ***Surgical technology RCTs*** | |  |  |  |  |  |  |  |
| Fahim 2022  (Netherlands) | Single-center | Lap/robotic AR with sponge assistance vs. Trendelenburg position | 150 (75/75) | Nov 2015-Jun 2021 | Age NR; Rectum (/Sigmoid); cStage I-IV | ≥10 sponge-assisted cases | Postoperative hospital stay | 11.3% |
| Jafari 2021  (USA) | Multicenter (25) | Anastomotic perfusion assessment after LAR with vs. without ICG | 347 (178/169) | Mar 2015-Feb 2017 | ≥ 18 years; Anastomosis ≤ 10 cm from AV; cStage I-III | ICG-trained surgeons | Anastomotic leak | 0% |
| Jayne 2025  (UK) | Multinational (8) multicenter (28) | Anastomotic perfusion assessment after lap/robotic AR with vs. without ICG | 766 (383/383) | Oct 2017-Aug 2023 | ≥18 years; AV ≤15 cm; cStage I-IV | ≥3 rectal cancer resections with ICG-guided anastomotic perfusion assessment | Anastomotic leak | 7.7% |
| Kneist 2023  (Germany) | Multicenter (8) | TME with vs. without piONM | 171 (82/89) | Feb 2013-Jan 2017 | 18-90 years; AV NR; cStage I-IV | Certified colorectal cancer center + piONM-trained surgeons | Urinary function | 0% |
| Teo 2023  (China) | Single-center | Rectal transection with lap vs. robotic stapler after robotic AR | 40 (20/20) | Mar 2018-Jul 2022 | ≥ 18 years; AV NR; cStage I-IV | ≥50 robotic cases; ≥50 stapler firings | Number of stapler cartridges | 0% |
| Tomasicchio 2024  (Italy) | Multicenter (5) | Anastomotic reinforcement after AR with vs. without cyanoacrylate | 133 (72/61) | Jun 2021-Jan 2023 | Age NR; AV 8-15 cm; cT2-4aNX | High-volume tertiary referral centers | Anastomotic leak | 0% |
| Wan 2022  (China) | Single-center | Lap AR with vs. without ICG-based LN mapping | 66 (33/33) | May 2021-Apr 2022 | 18-80 years; AV 5-30 cm; cStage I-III | Experienced surgeons trained by the principal investigator | Harvested D3 LNs | 0% |
| Watanabe 2023  (Japan) | Multicenter (41) | Anastomotic perfusion assessment after lap/robotic LAR with vs. without ICG | 839 (422/417) | Dec 2018-Feb 2021 | ≥ 20 years; AV ≤ 12 cm; cStage 0-III | Board-certified surgeons; > 30 lap rectal cancer resections | Anastomotic leak | NR |

China, People’s Republic of China; UK, United Kingdom; USA, United States of America; BMI, body mass index; PRO, patient-reported outcome; RCT, randomized controlled trial; AR, anterior resection; APR, abdominoperineal resection; AV, anal verge; NA, not applicable; MIS, minimally invasive surgery; LR, local recurrence; Lap, laparoscopic; TME, total mesorectal excision; DL, dentate line; DFS, disease-free survival; QoL, quality of life; HALS, hand-assisted laparoscopic surgery; NR, not reported; TaTME, transanal total mesorectal excision; OS, overall survival; SILS, single-incision laparoscopic surgery; LAR, low anterior resection; IMA, inferior mesenteric artery; CEMP, cephalomedial‐to‐lateral approach; MAP, medial‐to‐lateral approach; CSS, cancer-specific survival; DVF, Denonvilliers’ fascia; CRT, chemoradiotherapy; ISR, intersphincteric resection; SCRT, short-course radiotherapy; LE, local excision; TEM, transanal endoscopic microsurgery; TES, transanal endoscopic surgery; CAA, coloanal anastomosis; TCA, Turnbull-Cutait anastomosis; CRA, colorectal anastomosis; NOSE, natural orifice specimen extraction; ICG, indocyanine green; piONM, pelvic intraoperative neuromonitoring; LN, lymph node.

**Supplementary Appendixes**

**PRISMA 2020 Checklist**

| **Section and Topic** | **Item #** | **Checklist item** | **Location where item is reported** |
| --- | --- | --- | --- |
| **TITLE** | | |  |
| Title | 1 | Identify the report as a systematic review. | 1 |
| **ABSTRACT** | | |  |
| Abstract | 2 | See the PRISMA 2020 for Abstracts checklist. | 2-3 |
| **INTRODUCTION** | | |  |
| Rationale | 3 | Describe the rationale for the review in the context of existing knowledge. | 4-5 |
| Objectives | 4 | Provide an explicit statement of the objective(s) or question(s) the review addresses. | 4-5 |
| **METHODS** | | |  |
| Eligibility criteria | 5 | Specify the inclusion and exclusion criteria for the review and how studies were grouped for the syntheses. | 6-9 |
| Information sources | 6 | Specify all databases, registers, websites, organisations, reference lists and other sources searched or consulted to identify studies. Specify the date when each source was last searched or consulted. | 6-9 |
| Search strategy | 7 | Present the full search strategies for all databases, registers and websites, including any filters and limits used. | 6-9 |
| Selection process | 8 | Specify the methods used to decide whether a study met the inclusion criteria of the review, including how many reviewers screened each record and each report retrieved, whether they worked independently, and if applicable, details of automation tools used in the process. | 6-9 |
| Data collection process | 9 | Specify the methods used to collect data from reports, including how many reviewers collected data from each report, whether they worked independently, any processes for obtaining or confirming data from study investigators, and if applicable, details of automation tools used in the process. | 6-9 |
| Data items | 10a | List and define all outcomes for which data were sought. Specify whether all results that were compatible with each outcome domain in each study were sought (e.g. for all measures, time points, analyses), and if not, the methods used to decide which results to collect. | 6-9 |
|  | 10b | List and define all other variables for which data were sought (e.g. participant and intervention characteristics, funding sources). Describe any assumptions made about any missing or unclear information. | 6-9 |
| Study risk of bias assessment | 11 | Specify the methods used to assess risk of bias in the included studies, including details of the tool(s) used, how many reviewers assessed each study and whether they worked independently, and if applicable, details of automation tools used in the process. | 6-9 |
| Effect measures | 12 | Specify for each outcome the effect measure(s) (e.g. risk ratio, mean difference) used in the synthesis or presentation of results. | 6-9 |
| Synthesis methods | 13a | Describe the processes used to decide which studies were eligible for each synthesis (e.g. tabulating the study intervention characteristics and comparing against the planned groups for each synthesis (item #5)). | 6-9 |
|  | 13b | Describe any methods required to prepare the data for presentation or synthesis, such as handling of missing summary statistics, or data conversions. | 6-9 |
|  | 13c | Describe any methods used to tabulate or visually display results of individual studies and syntheses. | 6-9 |
|  | 13d | Describe any methods used to synthesize results and provide a rationale for the choice(s). If meta-analysis was performed, describe the model(s), method(s) to identify the presence and extent of statistical heterogeneity, and software package(s) used. | 6-9 |
|  | 13e | Describe any methods used to explore possible causes of heterogeneity among study results (e.g. subgroup analysis, meta-regression). | 6-9 |
|  | 13f | Describe any sensitivity analyses conducted to assess robustness of the synthesized results. | 6-9 |
| Reporting bias assessment | 14 | Describe any methods used to assess risk of bias due to missing results in a synthesis (arising from reporting biases). | 6-9 |
| Certainty assessment | 15 | Describe any methods used to assess certainty (or confidence) in the body of evidence for an outcome. | 6-9 |
| **RESULTS** | | |  |
| Study selection | 16a | Describe the results of the search and selection process, from the number of records identified in the search to the number of studies included in the review, ideally using a flow diagram. | 10-20 |
|  | 16b | Cite studies that might appear to meet the inclusion criteria, but which were excluded, and explain why they were excluded. | 10-20 |
| Study characteristics | 17 | Cite each included study and present its characteristics. | 10-20 |
| Risk of bias in studies | 18 | Present assessments of risk of bias for each included study. | 10-20 |
| Results of individual studies | 19 | For all outcomes, present, for each study: (a) summary statistics for each group (where appropriate) and (b) an effect estimate and its precision (e.g. confidence/credible interval), ideally using structured tables or plots. | 10-20 |
| Results of syntheses | 20a | For each synthesis, briefly summarise the characteristics and risk of bias among contributing studies. | 10-20 |
|  | 20b | Present results of all statistical syntheses conducted. If meta-analysis was done, present for each the summary estimate and its precision (e.g. confidence/credible interval) and measures of statistical heterogeneity. If comparing groups, describe the direction of the effect. | 10-20 |
|  | 20c | Present results of all investigations of possible causes of heterogeneity among study results. | 10-20 |
|  | 20d | Present results of all sensitivity analyses conducted to assess the robustness of the synthesized results. | 10-20 |
| Reporting biases | 21 | Present assessments of risk of bias due to missing results (arising from reporting biases) for each synthesis assessed. | 10-20 |
| Certainty of evidence | 22 | Present assessments of certainty (or confidence) in the body of evidence for each outcome assessed. | 10-20 |
| **DISCUSSION** | | |  |
| Discussion | 23a | Provide a general interpretation of the results in the context of other evidence. | 21-26 |
|  | 23b | Discuss any limitations of the evidence included in the review. | 21-26 |
|  | 23c | Discuss any limitations of the review processes used. | 21-26 |
|  | 23d | Discuss implications of the results for practice, policy, and future research. | 21-26 |
| **OTHER INFORMATION** | | |  |
| Registration and protocol | 24a | Provide registration information for the review, including register name and registration number, or state that the review was not registered. | 27 |
|  | 24b | Indicate where the review protocol can be accessed, or state that a protocol was not prepared. | 27 |
|  | 24c | Describe and explain any amendments to information provided at registration or in the protocol. | 27 |
| Support | 25 | Describe sources of financial or non-financial support for the review, and the role of the funders or sponsors in the review. | 27 |
| Competing interests | 26 | Declare any competing interests of review authors. | 27 |
| Availability of data, code and other materials | 27 | Report which of the following are publicly available and where they can be found: template data collection forms; data extracted from included studies; data used for all analyses; analytic code; any other materials used in the review. | 27 |

*From:* Page MJ, McKenzie JE, Bossuyt PM, Boutron I, Hoffmann TC, Mulrow CD, et al. The PRISMA 2020 statement: an updated guideline for reporting systematic reviews. BMJ 2021;372:n71. doi: 10.1136/bmj.n71. This work is licensed under CC BY 4.0. To view a copy of this license, visit <https://creativecommons.org/licenses/by/4.0/>
